# Comparison of Gold Yield with Traditional Amalgamation and Direct Smelting in Artisanal Small-Scale Gold Mining in Uganda

**DOI:** 10.5696/2156-9614-9.24.191205

**Published:** 2019-11-27

**Authors:** Birgitte Stoffersen, Rasmus Køster-Rasmussen, Jorge Ivan Contreras Cardeño, Peter W.U. Appel, Margrethe Smidth, Leoncio D. Na-Oy, Debbie Libua Lardizabal, Rudy W. Onos

**Affiliations:** 1 Diàlogos, Copenhagen, Denmark; 2 Department of Public Health, University of Copenhagen, Denmark; 3 Aalborg University, Aalborg, Denmark; 4 Small-scale miner, Itagon, Benguet, Philippines

**Keywords:** mercury, gold mining, small-scale mining, borax, amalgamation

## Abstract

**Background.:**

The amalgamation method used by artisanal small-scale miners is the single largest source of global mercury emission. The goal of the ‘Free Your Mine’ project is to stop mercury use in artisanal and small-scale mining.

**Objectives.:**

The aim of the present study was to compare gold recovery and time consumption between the amalgamation method and direct smelting, using borax for smelting under standardized conditions.

**Materials and Methods.:**

This was an experimental study in a pragmatic setting in the mining community of Tiira, Uganda. Standardized amounts of gold ore of equal quality were processed with the local amalgamation method and with the Philippine mercury-free method as practiced by miners from Benguet in the Philippines, and the gold yield and time consumption were compared.

**Results.:**

The amalgamation method took 53 minutes and recovered 1.0 g of pure gold. The miners used 4 g of mercury in the processing. The Philippine mercury-free method took 62 minutes and recovered 1.4 g of pure gold.

**Conclusions.:**

The Philippine mercury-free method recovered 40% more gold than the amalgamation method but took 9 minutes longer. The Philippine mercury-free method is a viable alternative to amalgamation.

**Competing Interests.:**

The authors declare no competing financial interests.

## Introduction

Artisanal and small-scale mining (ASGM) is the exploration, extraction and processing of metals deposits, precious stones and industrial minerals with predominantly simple, but high labor-intensive technologies. Artisanal small-scale gold mining is estimated to account for 20% of global gold production and is the source of income for approximately 15 million miners and provides livelihoods for about 100 million people.^1^ Artisanal small-scale gold mining is notorious for poor occupational safety in general and for the use of mercury in the process of recovering gold. Small-scale gold mining is estimated to contribute 37% of global mercury emissions.^2^ In order to stop this emerging environmental disaster and related health hazards, mercury-free methods must be implemented in ASGM communities worldwide. Towards this goal, most counties have signed the Minamata convention that aims to reduce global mercury pollution. Methods for gold extraction without the use of mercury are available. Direct smelting has proven to be safe and implementable in various ASGM communities.^3–7^

In January 2019, a team from the Danish non-governmental organization (NGO), Diálogos, the Ugandan NGO, National Association of Professional Environmentalists (NAPE) and a team of gold miners from the Philippines organized a mercury-free gold extraction workshop for a group of small-scale gold miners in Tiira in the district of Busia, Uganda. The main objective of the project was to limit mercury pollution by training local miners in direct smelting, so that they can continue to extract gold without using mercury and teach the method to their fellow miners. The objective of the present study was to compare gold recovery and time consumption of the locally used amalgamation method with direct smelting, which was taught in the course.

## Materials and Methods

This was an experimental study in a pragmatic setting. The training session was part of the three-year project ‘Free Your Mine’; a partnership between Diálogos and the NAPE, funded by the Civil Society in Development and the Danish Ministry of Foreign Affairs. The goal of the 3-year project was to implement mercury-free ASGM in Tiira and Buhweju using a civil society strategy involving not only miners, but also health care systems, schools, women's groups, and the political system and empowering the miners' own organizations. The miner trainers were from the Benguet province in the Philippines, where direct smelting is widely practiced.

The data are based on field observations by Diálogos staff during project visits in November 2018 and January 2019. The milling stations in the area were mapped with Global Positioning System and the workers were interviewed about their extraction methods and the use of mercury was observed.

The participants in training were voluntary ASGM workers from the Tiira area, selected by their ability to speak English. A fee of 20.000 Ugandan shillings, equal to approximately 5.5 USD, was paid to each participant per day of training to compensate for lost income. The participating miners were introduced to the health-related advantages of direct smelting during an informal lecture on mercury toxicology. All participants gave oral consent to participate in the present study. The Tiira Small Scale Miner Association hosted the training and the processing station used for the training was built by the NAPE and Diálogos for the program.

**Abbreviations**
*ASGM*: Artisanal and small-scale mining
*NAPE*: National Association of Professional Environmentalists
*NGO*: Non-governmental organization

**Figure 1:**
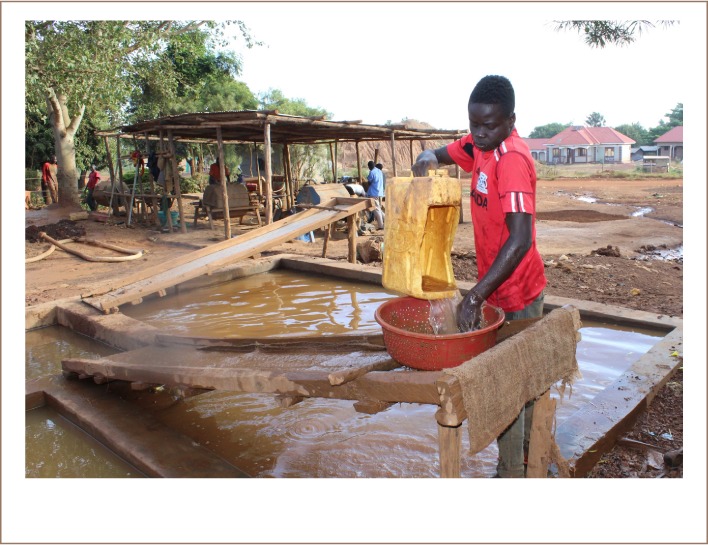
The local processing station in Tiira, Busia region, Uganda. A miner is washing the milled ore in order to collect the heavy minerals on the cloth (old sacks) covering the chute. Published with permission.

**Figure 2:**
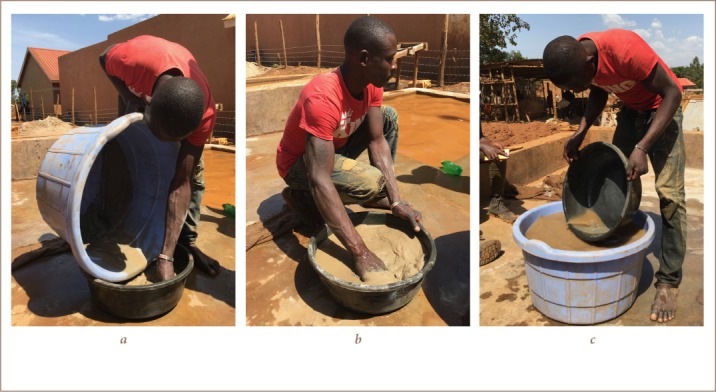
a) The loaded sacks are washed in a big tub and then transferred to the black washing tub. b) Mercury is added to the recovered material and mixed by hand. c) The light minerals are discarded by gravity (washing technique) little by little until only the amalgam is left. All images published with permission.

In Tiira village, 10 out of 10 milling stations used an amalgamation method and extracted gold using the following process: the gold-bearing ore was crushed followed by dry-milling into fine-grained powder in a metal ball mill. The powder was placed in a washing tub with small holes, where water was added, and the powder was washed down a chute covered with sacks that caught the heavy minerals including the fine-grained gold *(Figure 1)*. The sacks containing the heavy minerals were washed in a large tub, and the recovered material was put into a plastic tub with water, *(Figure 2a)*. Mercury (4 droplets worth 4000 UGX approximately equal to 1 USD) was added to the material in the tub and mixed by hand *(Figure 2b)*. Little by little, small portions of the lightest minerals were discarded from the tub by washing *(Figure 2c)*. The process was repeated until only the amalgam was left. The amalgam was then placed in a piece of textile and the excess mercury was squeezed out. The amalgam was placed on a spoon and burned with a Bunsen burner, which took only a few minutes. During the burning, most of the mercury evaporated, but a small fraction of mercury remained amalgamated with the gold.

### Direct smelting

**Figure 3:**
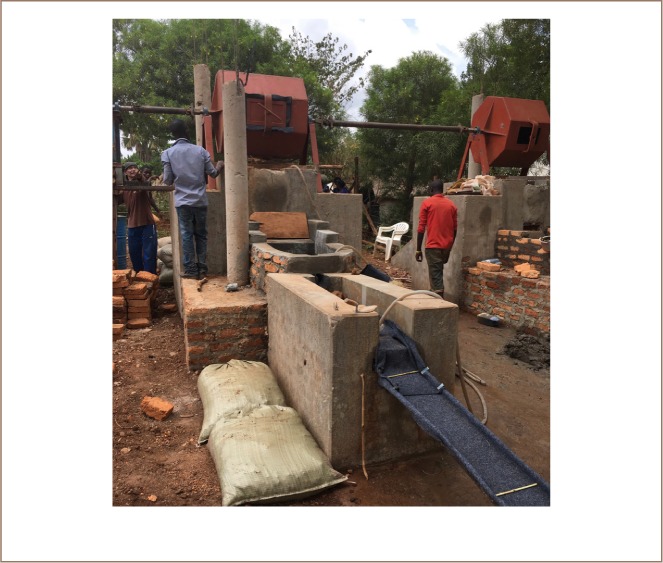
Processing station built as part of the project to process gold with direct smelting. Two interconnected ball mills followed by two successive sluice boxes and a launder. Published with permission.

In order to efficiently process gold-bearing ore without mercury, a processing station was built in Tiira on the land of the Tiira Small Scale Miner Association *(Figure 3)*. The miner trainers from the Philippines designed the facility, and local miners in Tiira adjusted it to their use. Technical drawings can be downloaded from Diálogos' homepage.^8^

In the Philippines, direct smelting borax is used for smelting gold. Borax is a sodium borate and is used as a flux during the smelting process of the gold, as it lowers the melting point of the gold to a temperature range that can be reached with simple technologies available in primitive settings.

During the direct smelting training course, the gold ore was wet milled, although during the comparison the gold ore was dry milled, as it is easier to mix and divide the ore equally. Except for the milling of the ore, the process was carried out both during the training course and the comparison as follows: the crushed gold ore was mixed with water and the wet mixture of fine-grained ore was washed through a sluice box and over the launder, covered with the textile felt. The tailings at the end of the launder were collected for reprocessing. When the felt was loaded with heavy minerals it was washed in a large tub, where soap was added in order to break the surface tension and make the small gold particles settle. The material recovered from the felt was then concentrated by traditional washing with a metal pan. Magnetite was removed with a magnet.

**Figure 4:**
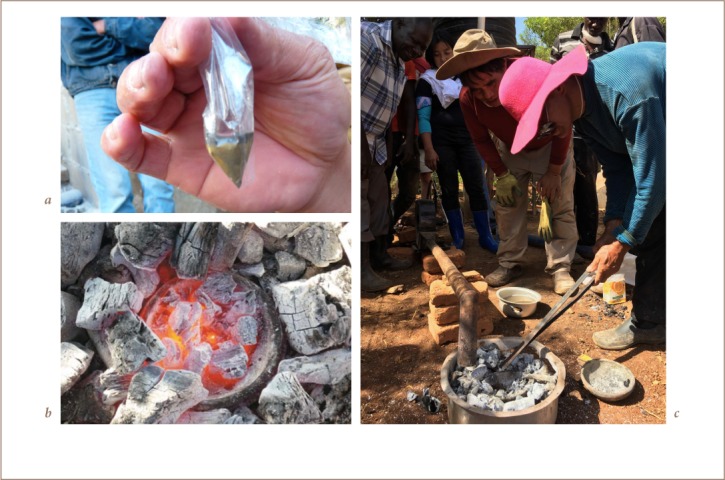
a) Plastic bag with gold concentrate, borax and a few drops of water. b) Clay ball with molten gold in the center. c) Smelting of the gold concentrate. Published with permission.

The mineral concentrate was ground with a stone in the pan in order to liberate fine-grained gold trapped in the mineral grains. The mixture was then panned further to concentrate the gold. The heavy mineral concentrate was placed in a tiny plastic bag and borax and a few drops of water were added *(Figure 4a)*. The amount of borax added was 30% of the most optimistic estimate of gold content; 1 g of borax was used, worth approximately 0.01 USD. The plastic bag, with gold concentrate and borax, was placed in a clay bowl covered with charcoal *(Figure 4b)*, which was then heated with a blower for 15 minutes *(Figure 4c)*. Another 1–2 g of borax was added during the heating.

### Comparison of the two methods

The comparison was carried out on the 27^th^ of January 2019. The purpose of the comparison was to compare the gold yield and efficiency of the two methods and to demonstrate this for the miners participating in the program.

**Figure 5:**
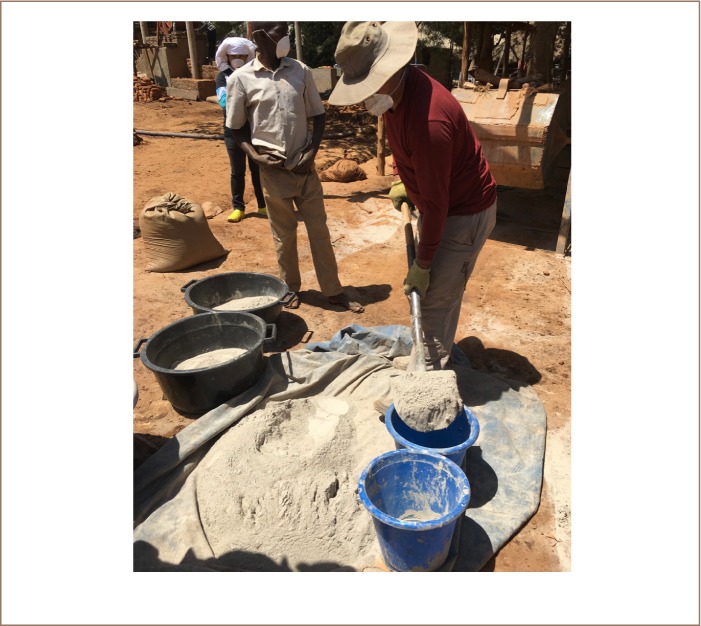
The dry milled ore is divided spade by spade into two lots. Published with permission.

To compare the methods, two uniform lots of ore of equal quality were processed; one for each method. It was assumed that the two lots contained equal amounts of gold due to the extensive mixing. All of the ore used for the comparison was dry milled, as dry milling makes it easier to accurately divide the material into two equal portions. The dry milled ore was then divided spade by spade into two equal portions, see Figure 5.

The efficiency of the methods was measured as the time it took for the miners to produce an amalgam ready to be burned and a gold concentrate in a plastic bag with borax and water ready to be burned. Subsequently, both the amalgam and the gold concentrate with borax were burned with borax in order to get a comparable measure of pure gold from both processes, as gold recovered with amalgamation and burned still contains a substantial fraction of mercury.

## Results

The amalgamation method took 53 minutes and recovered 1.0 g of pure gold. The miners used 4 g of mercury in the processing. Direct smelting took 62 minutes, recovered 1.4 g of pure gold and used no mercury. Hence, the gold yield was 40% higher with direct smelting than with the amalgamation method.

## Conclusions

Our experimental study in Tiira showed that direct smelting recovered 40% more gold than the amalgamation method but took 9 minutes longer.

The comparison was carried out in a setting that was familiar for the local miners using the amalgamation method, which may have biased the results in favor of the amalgamation method. Another limitation of the present study was that the comparison was carried out only once. More iterations would have made the results more accurate. Likewise, the results could have been more precise if the two lots of ore had been weighted out carefully, but the project was mainly an aid and technology transfer project and not much time could be allocated for carrying out scientific comparisons.

Another possible factor that might have biased the results in favor of the amalgamation method is that the ore was dry milled. Dry milling is extremely dusty. Thus, ore dust containing gold is lost to the environment during milling and unloading of the mill. Wet milling is normally used in direct smelting, but in this experiment dry milling was used for both methods for a thorough comparison. Dry milling likely resulted in a loss of a small amount of gold and thus biased the results in favor of the amalgamation method. The main downsides to dry milling are health-related complications, as inhaling the fine-grained dust may lead to silicosis. Furthermore, dry milling is much more noisy than wet milling, which may cause reduced hearing among the miners and people living close to the processing stations.

There are several possible reasons why the gold yield was higher with direct smelting. One is that mercury does not catch all of the very fine-grained gold, which is then lost to the environment.^5^ Another reason is that concentrating gold in a washing tub is not as effective as in a gold pan, and small grains of gold might be lost to the tailings. Direct smelting has an advantage when the gold ore contains large amounts of fine-grained gold. The efficacy is further improved by grinding in the pan and releasing even more fine-grained gold. Miners in the Philippines using direct smelting on a daily basis typically re-mill the tailings in order to liberate more fine-grained gold. In the present experiment, the ore was only milled and processed once, but further milling and processing would likely have resulted in a higher gold yield.

### Potential for implementation

Practicing direct smelting is more complex and slightly more time consuming than the amalgamation method. A phase-in period for adopting the new method requires time and practice. Formalization of direct smelting to the miners is an aspect that has shown to be very important in changing practices.^5^,^9^ The Diálogos strategy maintains that education of the local health care staff, political leaders, schoolteachers and children will create pressure on the miners to change their practices. Gaang, a mining community in the Philippines with 1200 ASGMs, changed to direct smelting and has now been mercury-free for seven years. In Gaang, a strong organization of miners facilitated this change, it was not the decision of an individual miner, but a common decision for all miners in the area.^5^ Therefore, empowerment and development of miner associations is a key feature in the ‘Free Your Mine’ project.

### Summary and recommendations

In the present study, direct smelting yielded 40% more gold than the amalgamation method, with a time consumption of 62 minutes and 53 minutes, respectively. The results contribute to the emerging body of evidence that direct smelting, as practiced by small-scale gold miners from the Benguet province in the Philippines, is a viable alternative to the amalgamation method. Future large-scale projects are warranted in order to comply with the Minamata convention, and we suggest direct smelting described in this article as a model to eliminate mercury use in ASGM.
